# Nanoparticle Delivery of Immunostimulatory Agents for Cancer Immunotherapy

**DOI:** 10.7150/thno.37216

**Published:** 2019-10-15

**Authors:** Jia Zhuang, Maya Holay, Joon Ho Park, Ronnie H. Fang, Jie Zhang, Liangfang Zhang

**Affiliations:** 1 Department of NanoEngineering, Chemical Engineering Program, and Moores Cancer Center, University of California San Diego, La Jolla, CA 92093, USA.; 2 Cello Therapeutics, Inc., San Diego, CA 92121, USA.

**Keywords:** biomimetic nanoparticle, cancer immunotherapy, immune stimulation, adjuvant, cytokine, checkpoint blockade

## Abstract

Immunostimulatory agents, including adjuvants, cytokines, and monoclonal antibodies, hold great potential for the treatment of cancer. However, their direct administration often results in suboptimal pharmacokinetics, vulnerability to biodegradation, and compromised targeting. More recently, encapsulation into biocompatible nanoparticulate carriers has become an emerging strategy for improving the delivery of these immunotherapeutic agents. Such approaches can address many of the challenges facing current treatment modalities by endowing additional protection and significantly elevating the bioavailability of the encapsulated payloads. To further improve the delivery efficiency and subsequent immune responses associated with current nanoscale approaches, biomimetic modifications and materials have been employed to create delivery platforms with enhanced functionalities. By leveraging nature-inspired design principles, these biomimetic nanodelivery vehicles have the potential to alter the current clinical landscape of cancer immunotherapy.

## 1. Introduction

The immune system, which is composed of different subsets of specialized immune cells, is highly efficient at eliminating exogenous material. The specific recognition of foreign antigens is mediated by professional antigen-presenting cells (APCs), which can present major histocompatibility complex (MHC)-restricted epitopes to T cells in the presence of costimulatory markers to promote both cellular and humoral immune responses 1, 2. While this process can be easily leveraged to effectively address infections caused by common pathogens, antitumor immunity is much more difficult to elicit. Although many tumor-associated antigens (TAAs) have been identified, they are generally lowly immunogenic 3. Tumors also develop a variety of mechanisms that enable them to subvert immune attack 4, 5. Through their ability to express immunosuppressive signaling molecules, modulate the functions of nearby immune cells, and change their phenotypes, tumor cells can escape from immune surveillance and continue to proliferate. Current cancer immunotherapies often work by rejuvenating the immune system in a manner that enables it to address the challenges associated with tumor immune escape, and many of these approaches have started to gain traction in the clinic 6. Whether they work by unleashing the functions of T cells 7, depleting immunosuppressive immune cell populations 8, or by modulating the characteristics of the tumor microenvironment 9, the common goal shared by most modern cancer immunotherapies is to augment endogenous immunity to ultimately overcome malignant growths.

**Figure 1:**
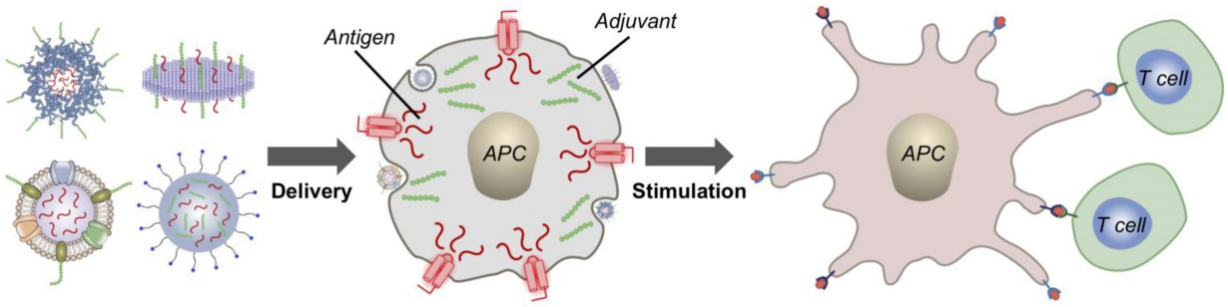
Delivery of immunotherapeutic payloads using biomimetic nanocarriers. Immunostimulatory agents such as adjuvants can be loaded along with antigenic material into biomimetic nanodelivery vehicles to enable enhanced delivery to specific immune cell subsets like antigen-presenting cells (APCs). Upon successful delivery, downstream immune processes such as T cell stimulation can be initiated to generate antitumor responses.

In general, the introduction of immunomodulatory compounds into the tumor microenvironment or surrounding immune-rich tissues is a promising means of elevating antitumor immunity. Here, we discuss the use of nanocarriers to enhance the delivery of these agents, which include adjuvants, secretory cytokines, and antibodies (**Figure 1**). Adjuvants are synthetic or naturally occurring compounds that are capable of activating pathogen recognition receptors (PRRs) found on APCs, thus generating strong proinflammatory responses 10. They can be administered along with antigenic material to generate potent tumor-specific responses and have also been explored as monotherapies capable of nonspecifically boosting immune activity. Cytokines are employed by a broad range of immune cells for signaling and communication and can exert immunomodulatory effects in complex ways 11. If used correctly, cytokines can directly stimulate immune effector cells at the tumor site and enhance tumor cell susceptibility to immune attack. Depending on the specific pathway being targeted, monoclonal antibodies (mAbs) can be used to antagonize immunosuppressive interactions or to promote immune stimulation 12. While adjuvants, cytokines, and mAbs all hold significant promise as anticancer therapeutics, these compounds can still benefit greatly from the increased specificity and enhanced safety afforded by nanodelivery platforms. In particular, emerging biomimetic technologies have the potential to provide improved functionality and to significantly enhance the potency of immunotherapeutic payloads, and these platforms will be covered in detail in this review.

## 2. Immunostimulatory Agents

### 2.1 Molecular Adjuvants

A number of different adjuvants that can stimulate the immune system are being developed and tested in clinical trials 13. One of the most popular targets for these compounds are Toll-like receptors (TLRs), which are expressed on APCs such as macrophages and dendritic cells (DCs) 14. TLRs have evolved to recognize specific molecular patterns from foreign microorganisms that act as danger signals to the immune system 15. TLR engagement can induce various gene expression profiles depending on the type of receptor and the type of stimuli, affecting both the innate immune response and adaptive immunity. One common target is TLR9, which can be activated by short single-stranded DNA with unmethylated CG motifs, referred to as CpG oligodeoxynucleotides (ODNs) 16. There are three classes of CpG ODNs, each of which has different biological activities 17. Some can be used as potent T helper cell type 1 (T_h_1)-biasing adjuvants and have shown great potential in cancer therapy. Another popular TLR target is TLR4, which can be activated by adjuvants such as lipopolysaccharides (LPS) 18. Because LPS exhibits significant toxicity, a less toxic derivative, monophosphoryl lipid A (MPLA), was developed by removing a phosphate residue. As a result of this modification, MPLA exhibits 1000-fold decreased toxicity compared to LPS and has been employed in some clinically explored vaccine formulations 19-21. Although LPS and MPLA both target TLR4, they can be associated with different cytokine secretion profiles 22.

Adjuvants that target other TLR pathways are also actively being researched. For example, poly(I:C) can activate TLR3 by mimicking viral RNAs 23. Poly(I:C) is a synthetic double-stranded RNA that has been extensively tested against diseases such as human immunodeficiency virus, dengue, malaria, and cancer. Since RNAs are inherently susceptible to degradation by RNase, poly(I:C) has been complexed with stabilizing molecules such as polylysine to prevent enzymatic degradation 24. Adjuvants that activate TLR5 include flagellin, which is a protein present in bacterial flagella 25. Flagellin alone can induce tumor necrosis factor-α (TNFα) production and can elicit high antibody titers when combined with vaccine antigens. Some imidazoquinoline derivatives with antiviral properties can activate TLR7 and TLR8 by mimicking single-stranded RNAs 26. For example, imiquimod (R837) activates TLR7 and resiquimod (R848) activates both TLR7 and TLR8, resulting in type I interferon (IFN) and interleukin-12 (IL12) production. R837 was approved by the United Stated Food and Drug Administration (FDA) and has been used in actinic keratosis 27, basal cell carcinoma 28, and genital warts 29 treatments.

Other targets for adjuvants include nucleotide-binding oligomerization domain (NOD)-like receptors and stimulator of interferon genes (STING) present on immune cells. NOD-like receptors regulate inflammation and innate immunity via inflammasomes 30. Synthetic adjuvants such as muramyl dipeptide can activate NOD2, which leads to the production of proinflammatory cytokines such as TNFα, IL1, IL6, and IL8 31. STING senses cyclic dinucleotides and nucleic acids of viral or bacterial origin 32. Activation of the STING pathway can lead to type I IFN secretion during infection 33. Cyclic di-AMP and cyclic di-GMP are cyclic dinucleotides originating from bacteria that have been used as STING agonists in vaccine development 34. These cyclic dinucleotides induce type I IFN and NF-κB-mediated cytokine production, helping to enhance antigen-specific T cell and humoral immune responses.

### 2.2 Cytokines

Cytokines are proteins employed in immune signaling, and they have been widely leveraged for their immunomodulatory effects 11. Many of the cytokines from the IFN and IL families are in clinical use or in clinical trials. IFNs are classified into three categories based on the receptors to which they bind. IFNα and IFNβ are popular examples of type I IFNs that are used as immune stimulating agents 35. IFNα has been approved by the FDA as an adjuvant therapy for stage III melanoma. The cytokine promotes MHC class I expression, which leads to better tumor antigen recognition. In preclinical cancer models, IFNβ has shown its potential as an immunostimulatory agent, as well as its ability to suppress autoimmune reactivity. However, it has yet to be applied in the clinic due to its low bioavailability and side effects. IFNγ is the only member of the type II IFNs 36. It promotes MHC expression in macrophages and induces the expression of costimulatory molecules on APCs. IFNγ can also promote the T_h_1-biased differentiation of CD4^+^ T cells and inhibit IL4-dependent isotype switching in B cells. Type III IFNs, which include the IFNλ group of molecules, are relatively new compared to type I or type II IFNs 37. Although it is known that IFNλ plays a role in certain antiviral immune responses, its potential as an immunostimulatory therapeutic has yet to be fully explored.

Among the ILs, IL2 has been approved by the FDA for use in treating metastatic melanoma 38 and renal cell carcinoma 39. IL2 promotes the activation and expansion of CD4^+^ and CD8^+^ T cells, as well as the proliferation of natural killer (NK) cells. Not only does IL2 activate immune responses, but it can also act as a mediator of immune tolerance by suppressing T cell responses 40. Another clinically relevant IL is IL12, which acts as a growth factor for activated NK and T cells and promotes production of IFNγ 41. IL12 can also help CD4^+^ T cells to differentiate into a T_h_1 phenotype and increases the activity of CD8^+^ cytotoxic T lymphocytes (CTLs). Although IL12 has gone through various preclinical investigations and showed anti-angiogenic efficacy mediated by IFNs, it has yet to be translated.

### 2.3 Monoclonal Antibodies

Apart from adjuvants and cytokines, mAbs represent another means of achieving immune modulation. They offer certain advantages, including high specificity, resistance against degradation in serum, and long circulation times 42. As immunostimulatory agents, mAbs can specifically activate (agonistic mAbs) or suppress (antagonistic mAbs) certain cellular pathways, making them a compelling tool to explore 43. Anti-CD28 can stimulate immune responses by interacting with its target, which is constitutively expressed on most resting CD4^+^ T cells and a significant portion of CD8^+^ T cells 44. This agonistic interaction triggers signaling cascades that promote proliferation, cytokine production, anti-apoptotic gene expression, and energy metabolism. In most cases, anti-CD28 mAbs cannot work alone and their use must be accompanied by antigen-dependent T cell receptor (TCR)-mediated signals in order to properly activate T cells. 4-1BB, also known as CD137, can be found on T cells, NK cells, DCs, mast cells, and sometimes endothelial cells of metastatic tumors 45. Use of anti-4-1BB to engage this receptor triggers signaling pathways that lead to increased expression of anti-apoptotic genes. Similar to 4-1BB, OX40 is another member of the TNF receptor superfamily, and anti-OX40 mAbs can be used to stimulate CD4^+^ and CD8^+^ T cells 46. Activation of OX40 signaling in T cells can lead to enhanced proliferation and increased cytokine production. Another important TNF receptor is CD40, which is expressed on, but not limited to, B cells, DCs, macrophages, T cells, vascular endothelium, and some types of cancer cells 47. CD40 ligation is crucial in the humoral immune response and anti-CD40 mAbs can be used to stimulate antitumor activity. A prominent mechanism of this antitumor activity is the activation of the antigen-presenting DC network. Lastly, glucocorticoid-induced TNF receptor (GITR) is a costimulatory molecule that is expressed on activated T cells 48. Anti-GITR can activate GITR to increase the proliferation, activation, and cytokine production of CD4^+^ and CD8^+^ T cells.

Antagonistic mAbs can be used to downregulate or disrupt certain immune pathways that promote tumor growth 49. Checkpoint blockade therapies based on this type of approach have experienced a significant amount of success in clinical settings 50. PD-1, which is a member of the CD28 family, is a co-inhibitory receptor and is upregulated when CD4^+^ T cells, CD8^+^ T cells, B cells, and monocytes are activated 51. Engagement with its ligand, referred to as PD-L1, inhibits T cell activation and proliferation, causing cell-cycle arrest but not apoptosis. The use of anti-PD-1 and anti-PD-L1 mAbs to recover CTL-mediated antitumor effects is an approach that has been widely explored in the clinic. Similarly, cytotoxic T lymphocyte-associated protein 4 (CTLA-4) is also homologous with the costimulatory receptor CD28 52. CTLA-4 protein expression is upregulated when T cells interact with presented versions of their cognate antigens, and this in turn leads to a decrease in T cell activation. One of the most notable mechanisms by which CTLA-4 achieves T cell inhibition is by outcompeting CD28 for ligand binding, thus decreasing costimulation. In terms of cytokines, IL10 can be a compelling target since one of its main roles is to help avoid excessive immune activation, such as in autoimmune diseases 53. IL10 is produced by various myeloid and lymphoid cells and it suppresses macrophage and DC function, which leads to decreased activity and cytokine production. High levels of IL10 can lead to various pathologies, and IL10 antagonists have the potential to be used against chronic infection or cancer. Other novel immune checkpoint markers, including lymphocyte-activation gene 3 54, T cell immunoglobulin- and mucin-domain-containing molecule 3 55, T cell immunoreceptor with immunoglobulin and ITIM domains 56, V-domain immunoglobulin-containing suppressor of T cell activation 57, and B7/H3 58, are also actively being investigated.

## 3. Current Delivery Strategies

### 3.1 Benefits of Particulate Delivery

Despite their promise as therapeutics, immunostimulatory agents usually suffer from suboptimal pharmacokinetics, vulnerability to biodegradation, and compromised cell targeting when directly administered into the body 59. Their nonspecific interactions with proteases, nucleases, and immune cells not only reduce immunostimulatory capacity, but can often result in safety concerns and lead to excessive inflammation, toxicity, and hypersensitivity 60. Thus, there has been high demand for methods to effectively deliver immunostimulants to their target cell populations with minimal exposure to the surrounding biological environment. Emerging delivery strategies based on nanoparticle platforms offer an effective means of addressing the underlying issue, whereby payloads are complexed with biocompatible nanomaterials 61. The formulation of immunostimulatory payloads into nanocarriers can help to improve immune tolerance throughout the transport process, while also enhancing immune stimulation upon delivery to the appropriate immune cells.

The nanodelivery of immunostimulatory agents offers several benefits compared with use of the same compounds in their free form. First, payload entrapment and protection by a nanoparticle matrix minimizes the chance of interference caused by degradative agents and nonspecific cellular interactions 62. This helps to prolong circulation half-life and enhances the biological stability of the payload, both of which are crucial for maximizing downstream immune stimulation. Second, owing to the relatively small size of nanocarriers, the encapsulated payloads can more readily localize and accumulate at tumor sites or immune-rich tissues via common administration routes. For example, the subcutaneous administration of nanocarriers enables efficient transport to the draining lymph nodes, where the resident immune cells can be readily manipulated 63, 64. Furthermore, targeting capability towards specific immune cell populations can greatly enhance the efficacy of immunostimulant delivery, since most immunostimulatory agents act on specific pathways that are only relevant to certain cell subsets 65. By leveraging proper materials design, nanoparticulate platforms can be synthesized with specific targeting functionality and controllable release to greatly improve payload bioavailability and ensure immune activation at minimal dosages of the active ingredient 66, 67. A final advantage of nanocarriers is their ability to co-deliver immunostimulants and antigens together using the same particulate platform, which can improve the antigen presentation process and lead to better T cell stimulation 2, 68.

### 3.2 Current Delivery Platforms

#### 3.2.1 Polymers

**Figure 2:**
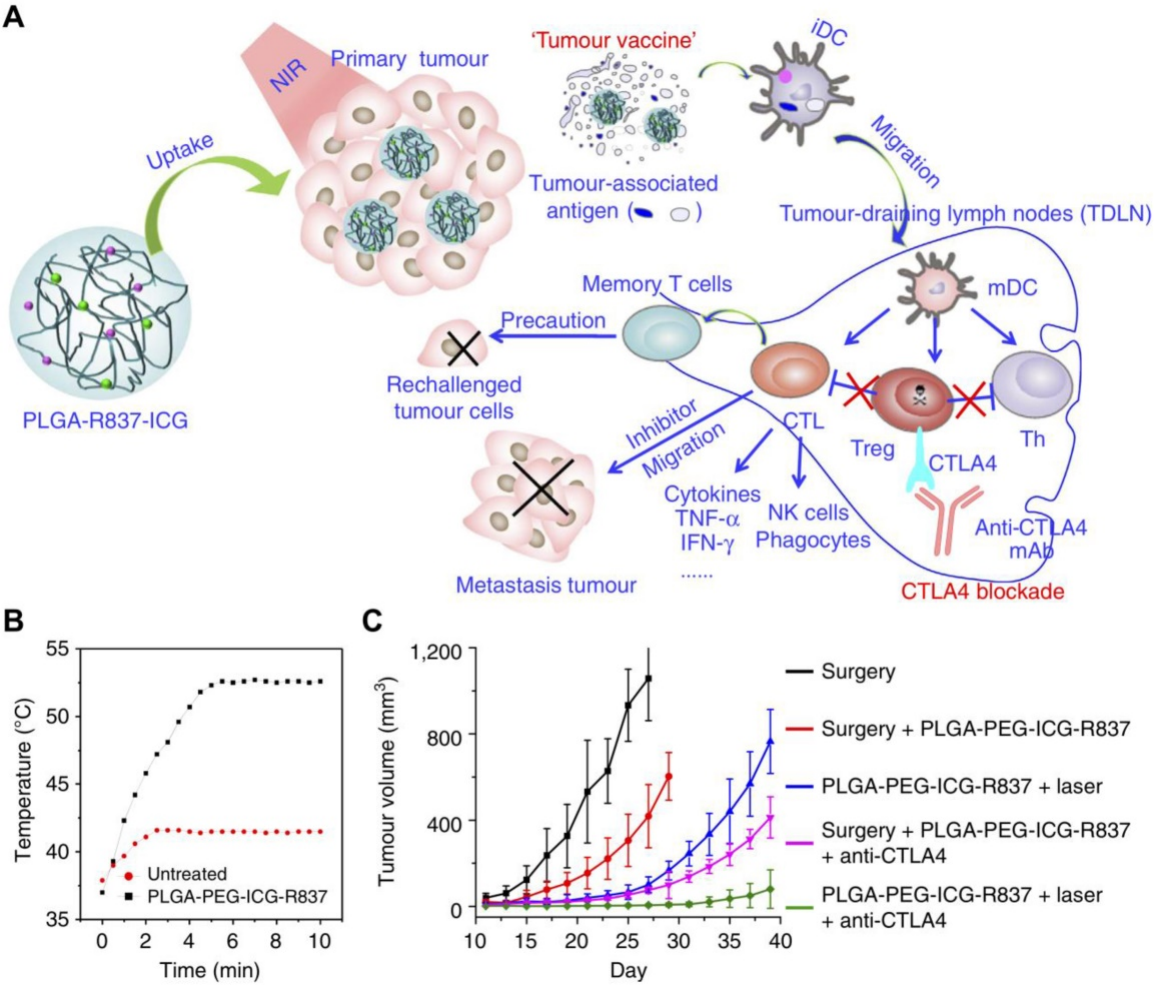
Adjuvant delivery using polymeric nanoparticles for combination therapy. (A) Poly(lactic-*co*-glycolic acid) (PLGA) nanoparticles loaded with R837 and indocyanine green (ICG) can be used to generate tumor antigens and promote transition of DCs from an immature (iDC) to mature (mDC) phenotype. The antitumor effect can further be enhanced through the inclusion of checkpoint blockades such as anti-CTLA-4. (B) The nanoformulation can be used to induce drastic temperature changes at tumor sites upon irradiation. (C) Photothermal therapy together with CTLA-4 blockade delays the growth of secondary tumors. Adapted with permission from 70. Copyright 2016 Nature Publishing Group.

Polymeric carriers represent one of the most prevalent and well-studied immunostimulant delivery vehicles. Polymers offer a wide range of conjugation and encapsulation options, and many have excellent biocompatibility profiles that make them a safe option for immunotherapy. Additionally, nanoscale polymeric delivery systems have the inherent ability to improve cancer immunotherapy because of their tendency to accumulate in tumor sites via the enhanced permeation and retention (EPR) effect 69. Polymeric platforms have been widely used for the delivery of adjuvant payloads. For instance, R837, along with a near-infrared dye, were co-encapsulated into a polyethylene glycol (PEG)-poly(lactic-*co*-glycolic acid) (PLGA) nanoparticle via an oil-in-water emulsion (**Figure 2**) 70. Here, the photothermal therapy component of the platform acted not only as a means of reducing tumor cell counts, but also primed the site for immune activity by generating tumor antigens for immune cell uptake. While free adjuvants in general cannot specifically accumulate into tumors, the R837-loaded polymeric nanoparticles benefited from the EPR effect and showed preferential tumor accumulation after intravenous injection. When administered in combination with anti-CTLA-4 mAbs, which helped to reverse the immunosuppression caused by regulatory T cells, the nanoformulation greatly inhibited the growth of secondary tumors, and mice were resistant to re-challenge, proving the long-term memory effects of the treatment.

A promising immunotherapeutic approach has been to combine checkpoint inhibitor treatment together with local DC activation using adjuvants 71. While the direct administration of adjuvants that are capable of activating DCs by triggering their PRRs have been explored, severe adverse effects and expedited clearance have limited the clinical application of this strategy 72. To improve translational potential, nanocarriers based on a block copolymer made of methoxytriethyleneglycol methacrylate and pentafluorophenyl methacrylate have been functionalized with TLR7/8 agonists capable of locally activating DCs in the tumor site 73. When combined with checkpoint blockades, the combination treatment was able to stall tumor growth in a B16 melanoma mouse model by eliciting DC activation and subsequent antitumor immunity. On a similar note, adjuvants can be useful agonists for the maintenance of antitumor activity after tumor resection. Because post-operation healing can often promote metastasis 74, maintaining an immunostimulatory microenvironment at the tumor site is critical.

**Figure 3:**
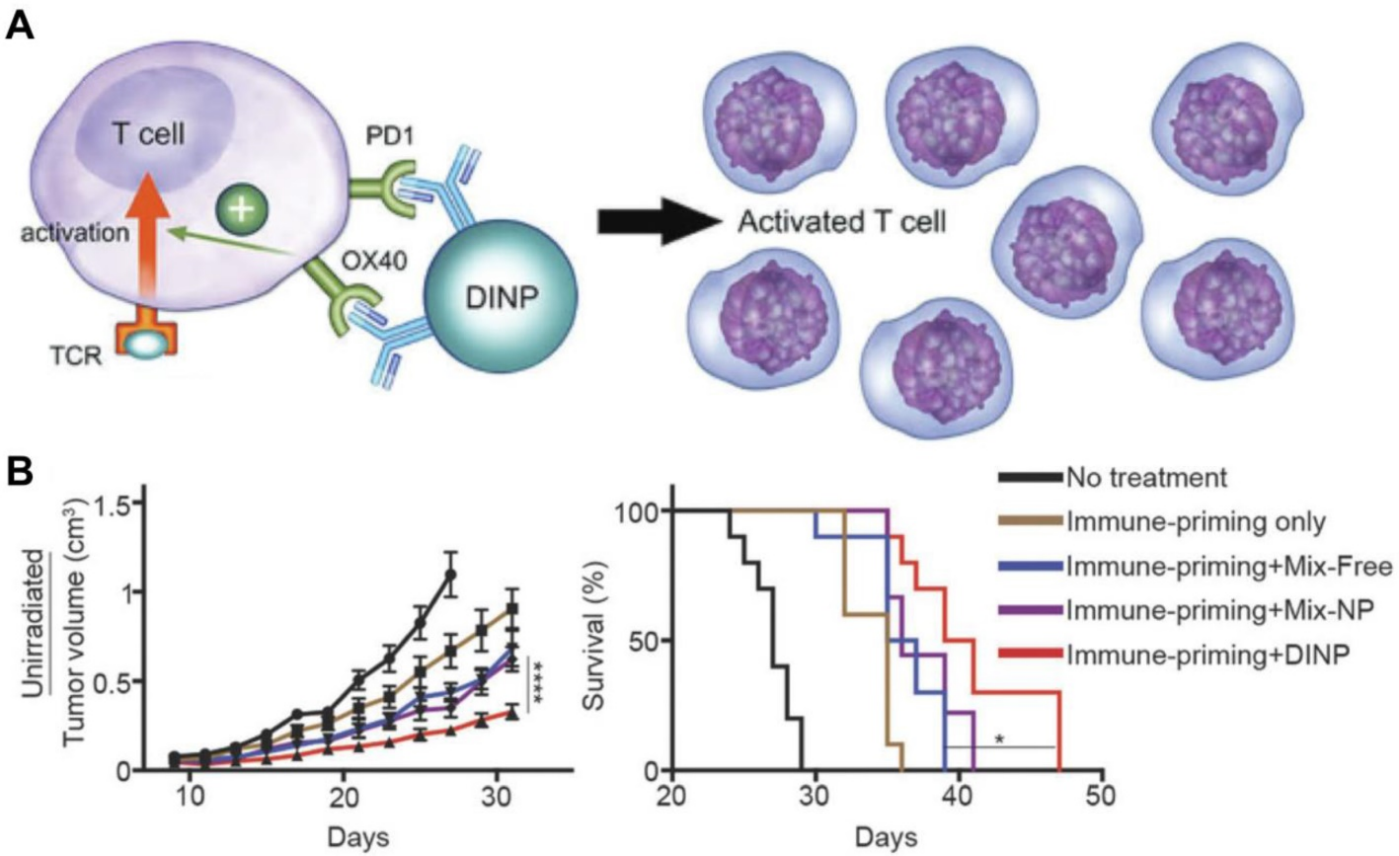
Dual delivery of antibodies for immune stimulation. (A) Anti-PD-1 and anti-OX40 mAbs can be co-delivered using a dual immunotherapy nanoparticle (DINP) design. The anti-PD-1 acts as an antagonist that reverses T cell exhaustion, while the agonistic anti-OX40 further promotes cell activation. (B) The DINP formulation improves the efficacy of combination immunotherapy *in vivo* . Adapted with permission from 78. Copyright 2018 Wiley-VCH.

OX40 is an important TNF receptor on the surface of some activated immune cells and helps to regulate, among others, both CD4^+^ and CD8^+^ T cells 75. Engagement of OX40 leads to proinflammatory cytokine production and T cell expansion; however, clinical trials using anti-OX40 mAbs have shown that the nonspecific nature of this immune activation makes it ineffective against lowly immunogenic tumors 76. As a result, better antibody delivery systems capable of increasing T cell priming and immune cell exposure are of great need. In one example, anti-OX40 mAbs were attached to PLGA nanoparticles by chemical conjugation onto the surface 77. These antibody-conjugated polymeric nanoparticles promoted increased proliferation and activation of CTLs *in vitro* when compared to mAbs alone, demonstrating the advantages of the nanoparticulate formulation. To add additional biological functionality, an antagonist antibody capable of blocking checkpoint inhibitors has also been conjugated onto the nanoparticle surface 78. A combination of anti-PD-L1 and anti-OX40 were attached onto PEGylated PLGA nanoparticles via thiol-maleimide chemistry (**Figure 3**). With both antibodies conjugated onto the same nanoparticle surface, T cells could interact with them simultaneously, thereby increasing activation, efficacy, and memory functionalities. Improved immunotherapeutic responses compared with a free antibody mixture or single-antibody formulations were demonstrated in two murine models, highlighting the benefits of presenting both checkpoint inhibitors and immunostimulatory antibodies together on the same nanoparticle.

Acetalated dextran has recently been shown to have properties that can be used to modulate various immunological pathways, making it an good material for developing cancer immunotherapies 79. Due to its highly tunable degradation rate, different versions of the polymer can be used to promote antigen cross-presentation through either transporter associated with antigen processing (TAP)-dependent or TAP-independent pathways. In addition to its pH-responsive and biodegradable properties, acetalated dextran is better than traditional polymer systems in its ability to efficiently load hydrophilic drugs 80. In one study, it was shown that acetalated dextran microparticles encapsulating either CpG ODN or poly(I:C) had higher loading efficiencies and elicited stronger *in vitro* immune responses when compared to their PLGA counterparts 81. Being pH-sensitive, acetalated dextran dissolves quickly under acidic conditions but remains stable at physiological conditions. This property can be taken advantage of in order to enhance adjuvant delivery to TLR receptors that reside in the acidic lysosomal compartments of APCs.

#### 3.2.2 Liposomes

Liposomes represent a popular choice for improving the biocompatibility and therapeutic lifetime of immunostimulatory agents. Payloads can be conjugated onto the liposomal membrane or loaded into the center, either directly or via an inner core material around which the liposome is coated. Recent efforts have taken advantage of liposomal carriers to deliver various immunostimulants to enhance their immune activating properties 82, 83. A major clinical limitation of the direct use of cytokines and mAbs is their systemic toxicity, specifically on circulating lymphocytes. To overcome this challenge, nanoscale particles have been leveraged for their passive targeting capabilities to more specifically deliver these agents to tumor sites. In one recent example, PEGylated liposomes with IL2 and anti-CD137 mAbs were fabricated 84. The immunostimulatory liposomes had remarkable tumor accumulation and improved anti-CD137 mAb and IL2 localization compared with their soluble forms. Ultimately, the formulation was successful in delaying tumor growth without adverse effects, indicating an improved safety profile.

#### 3.2.3 Emulsions

Oil-in-water emulsions have demonstrated the ability to positively modulate immune responses, and their use as adjuvants has achieved clinical success 85. Among other immune stimulation mechanisms, their ease of deformation allows for the lateral movement of antigens, which can enhance uptake and activation in APCs. More recent oil-in-water emulsion platforms have incorporated additional payload molecules to further improve immunotherapeutic potential. For example, polymer-squalene emulsions loaded with CpG ODN and model antigens have been used to generate antigen-specific T cell responses and promote tumor regression 77, 86, 87. Alternatively, water-in-oil emulsions can also provide immunostimulatory properties, although the effects are generally more localized to the site of injection. In one instance, anti-CTLA-4 antagonistic mAbs and anti-CD40 agonistic mAbs were loaded into water-in-oil emulsion microparticles 88. Due to the large size of the particles, these water-in-oil microemulsions provided a depot for localized and sustained therapeutic release when injected adjacent to the tumor site.

#### 3.2.4 Hydrogels

Nanosized hydrogels, or nanogels, have been recognized as an excellent type of material for biomolecule delivery. They have certain advantages over other nanocarriers and are particularly well-suited for biomolecule encapsulation 89. Nanogels can be made by the self-assembly of amphiphilic polysaccharides, and platforms based on cholesterol-bearing pullulan (CHP) have been studied for cancer immunotherapy applications 90. In one example, CHP nanogels were shown to drain to nearby lymph nodes upon subcutaneous administration, efficiently delivering their tumor antigen payload to APCs and eliciting strong antitumor immunity 91. Even without the co-administration of adjuvants, CHP nanogel TAA formulations have been shown to elicit both cell-based and antibody responses 92. Other nanogel systems have also been reported for cancer immunotherapy. For instance, a bioreducible cationic alginate-polyethylenimine nanogel was used to encapsulate ovalbumin (OVA), and the resulting nanovaccine was readily taken up by DCs, which enabled presentation of the antigen to lymphocytes for eliciting both humoral and cellular immune responses 93. To provide additional immune stimuli to nanogel systems, adjuvants can be crosslinked into the particle matrix. In an example using CpG ODN with a β-glucan nanogel, the resulting formulation induced much stronger antigen-specific T_h_1 responses than β-glucan nanogel alone 94. Specifically, mice preimmunized with an adjuvanted and antigen-loaded formulation exhibited a long delay in tumor growth and improved survival after tumor inoculation.

**Figure 4:**
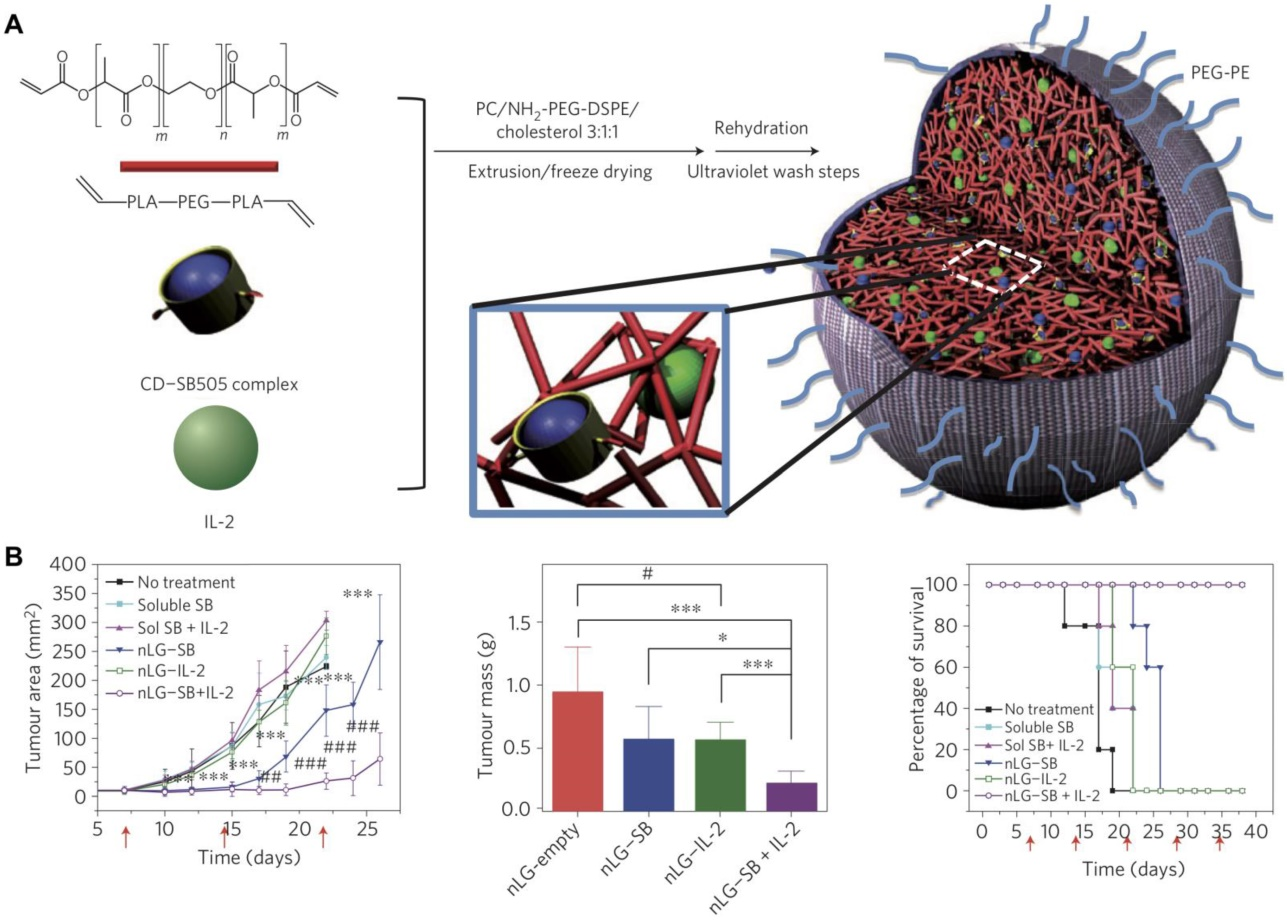
Dual delivery of immunostimulatory payloads using liposomal polymeric nanogels. (A) IL2 and a TGFβ inhibitor, SB505, complexed with cyclodextrin (CD) are loaded inside a biodegradable polymer hydrogel and coated with liposomal material to form a nanolipogel (nLG). (B) The dual-loaded nLG formulation enables significant control of tumor growth and extends survival in a cancer model. Adapted with permission from 97. Copyright 2012 Nature Publishing Group.

In addition to adjuvants, cytokines can also be incorporated into nanogels. For example, recombinant murine IL12 was successfully incorporated into a CHP nanogel through simple incubation at room temperature 95. After subcutaneous administration, the nanogel enabled the sustained release of IL12 into the bloodstream, which led to a prolonged elevation in IL12 serum levels. Repetitive administration of the formulation drastically retarded the growth of tumors without any apparent adverse effects. In another work, IL12 was encapsulated inside a modified CHP nanogel using a thiolated PEG as a crosslinker 96. The formulation hydrolytically degraded under physiological conditions, which resulted in the prolonged release of IL12 over time. After subcutaneous administration in mice, high IL12 levels were detected in the plasma. A nanosized core-shell liposomal polymeric gel has been developed for the co-delivery of a hydrophobic drug and a hydrophilic cytokine in the same system 97. Methacrylate-conjugated β-cyclodextrin was used to solubilize a transforming growth factor-β (TGFβ) inhibitor, and the drug-complexed β-cyclodextrin was then co-loaded inside a liposome shell along with IL2 and a biodegradable cross-linker (**Figure 4**). After photopolymerization, the formed hydrogel was able to deliver the two payloads into the tumor microenvironment in a sustained fashion. The release of the TGFβ inhibitor and IL2 significantly delayed tumor growth by promoting NK cell activation and CD8^+^ T cell infiltration in a murine B16F10 melanoma model.

#### 3.2.5 Gold Nanoparticles

Overall, gold nanoparticles (AuNPs) are accepted as a promising delivery platform due to their relative safety and tunable nature 98. They can also increase the potency and decrease the toxicity of immunotherapeutics due to enhanced accumulation in tumor sites via the EPR effect. In an example, AuNPs were used as substrates for multilayer coatings made by the layer-by-layer assembly of immune signals 99. Built through electrostatic and hydrophobic interactions, this polyelectrolyte self-assembled formulation contained poly(I:C) adjuvant and peptide antigens. Similar to other nanovaccine platforms, the co-delivery of adjuvant and antigen acted synergistically to provide greater expansion of CD8^+^ T cells when compared to immunization with a simple mixture of the components. The AuNP core also provided an appropriately sized substrate to aid in efficient uptake by APCs. The introduction of active targeting moieties can further improve potency and safety, offering the opportunity for active cytokine delivery without systemic toxicity. For example, AuNPs conjugated with a tumor homing peptide that recognizes and binds to CD13 on tumor endothelium were shown to effectively carry and release TNF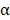 *in vivo* 100. Notably, administration of free cytokine at the same dosage showed no activity, highlighting the benefits of nanodelivery.

AuNPs may provide additional functionalities to antibody-based cancer immunotherapy, particularly given their ability to be used as contrast agents for computed tomography (CT) imaging and as transducers for photothermal therapy. When conjugated with checkpoint inhibitors, AuNPs can be made into theranostic platforms. In one example, anti-PD-L1-conjugated AuNPs were administered to tumor-bearing mice 101. When the mice underwent a CT scan, the signal correlated well with tumor growth and T cell infiltration, providing evidence that the formulations could be effectively used to predict treatment outcomes. In addition to CT imaging, AuNPs also exhibit surface plasmon resonance in the near-infrared range, thus enabling their use for photothermal therapy in combination with chemoimmunotherapy 102.

#### 3.2.6 Mesoporous Silica

Mesoporous silica nanoparticles (MSNs) have been studied in the field of nanomedicine. Unlike conventional aluminum adjuvants, MSNs can be easily doped with components that can improve their biodegradability and biocompatibility profiles 103, 104. Owing to the intrinsic high payload encapsulation capacity afforded by their porous structures, MSNs can act as delivery vehicles for a variety of immunostimulatory agents. In the case of adjuvants, combination therapies based on MSNs appear to be an effective approach. In one example, liposome-coated MSNs were loaded with doxorubicin and oxaliplatin as apoptosis inducers along with indoximod, an immunometabolic adjuvant that can interfere with immunosuppressive pathways in the tumor microenvironment 105. These particles benefited from increased circulation half-life and passive tumor targeting due to their biocompatible nature and nanoscale size. In a luciferase-expressing orthotropic pancreatic cancer model, tumor growth was significantly controlled with this combination therapy, and antigen-specific CTLs were clearly present.

MSNs may also provide a platform for reducing the systemic toxicity of encapsulated payloads, a necessity for the clinical use of many cytokines. For instance, the biologically active dosage of TNF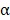 is one order of magnitude higher than the maximal permitted dosage for intravenous administration 106. To overcome this hurdle, MSNs can be functionalized to shield and control TNF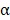 delivery. In an example, MSNs were fabricated with a pH-sensitive copolymer that acted as a gatekeeper 107. This platform enabled high drug loading in the mesopores of the MSNs and localized release, which was facilitated by the acid-triggered degradation of the copolymer. It has also been shown that mesoporous silica itself can act as a costimulant, provoking T_h_1 immunity and inducing both primary and memory immune responses 108. Its adjuvancy is heavily dependent on size and porosity. While maintaining high loading capacity and biocompatibility, large-pore MSNs capable of inducing strong immune responses when combined with photothermal agents and model antigens have been fabricated 109. Importantly, when compared directly to their silica counterparts, the MSNs generated a higher frequency of CD4^+^ and CD8^+^ T cells, highlighting the adjuvanting properties of particles. In a final example of MSN usage, biodegradable glutathione-depleted dendritic mesoporous organosilica nanoparticles were loaded with a model antigen and CpG ODN 110. Here, not only were the MSNs able to deliver their contents intracellularly, but they were also used to neutralize intracellular glutathione, leading to an excess generation of reactive oxygen species that served to further intensify immune responses.

## 4. Biomimetic Delivery Strategies

### 4.1 Introduction to Biomimetic Delivery

Particulate delivery systems have demonstrated the ability to enhance the bioavailability of immunostimulants and can promote increased immune activation; however, conventional platforms can still be limited by certain pitfalls. For instance, in spite of effective incorporation into delivery systems, some of these immunostimulatory agents still need to be delivered in large quantities to achieve the desired effects, which necessitates the use of delivery platforms with high loading yields 111. Finding alternative solutions to achieve better immune stimulation at lower dosages would thus be highly beneficial. Another challenge with many conventional delivery platforms is that they are still regarded as foreign by the immune system, which can lead to rapid immune clearance or unwanted immune responses 112. Furthermore, delivery of immunostimulant payloads to the appropriate immune cell populations is essential for proper immune activation. As such, targeted delivery approaches could ensure better immune recognition and augment overall immune responses 113.

An ideal immunostimulant delivery platform would interact minimally with irrelevant cells but elicit strong immune stimulation upon reaching target immune cells 114. As a result, on-demand immune activation could be achieved without compromised safety or tolerability parameters. Recently, biomimetic nanodelivery platforms have been increasingly employed for the delivery of immunostimulatory agents because of their ability to readily fulfill some of these design requirements 115-118. Biomimetic modifications or delivery vehicles have the potential to significantly improve upon the overall delivery efficiency and subsequent immune responses associated with current delivery platforms. In this section, three general approaches for achieving biomimetic delivery will be discussed in depth (**Table 1**).

### 4.2 Biomimetic Modifications

Biological targeting functionality can be achieved by employing naturally occurring moieties to modify the surface of nanoparticles, thus enhancing uptake efficiency by target immune cells. These modifications are oftentimes achieved through chemical conjugation or physical incorporation processes that are easy to implement and highly controllable 66. One representative ligand is mannose, which has affinity to receptors that are abundant on APCs 119. Mannose receptors on macrophages and DCs enhance affinity towards the cell surface of microorganisms, facilitating their uptake and subsequent presentation to T cells 120. When mannose is attached as a targeting ligand to immunostimulant delivery platforms, these mannosylated vehicles can be readily recognized and internalized by APCs, resulting in enhanced immune stimulation. In one example, a vaccine delivery system based on mannosylated chitosan microspheres was formulated for intranasal mucosal vaccination 121. Compared to unmodified particles, the mannosylated microspheres could tightly bind with mannose receptors on murine macrophages and stimulated immunoglobulin production. Similarly, a PEG-sheddable, mannose-modified polymeric nanoparticle platform has been assembled and shown to efficiently target tumor-associated macrophages after PEG shedding in the acidic tumor microenvironment 122. In a case of DC targeting, mannose was used to modify lipid-calcium phosphate nanoparticles, which contained the Trp2 melanoma self-antigen and CpG ODN as an adjuvant for immunotherapy against melanoma 123, 124.

Mannosylation can help to enhance nanoparticle localization in the lymph nodes, facilitating antigen presentation by DCs. In an example, mannose was selected to decorate chitosan nanoparticles 125. Due to the innate immunostimulatory effect of chitosan, the nanoparticles were able to elicit strong immune responses without the addition of any other immunostimulants. The mannose-modified chitosan nanoparticles were loaded with whole tumor cell lysate prepared from B16 melanoma cells. Prompt uptake by endogenous DCs within the draining lymph node was observed, which correlated with an elevation in IFNγ and IL4 levels. The therapeutic effects of this formulation were remarkable and resulted in a significant delay of tumor growth in an animal model of melanoma.

DC targeting can also be achieved using other sugar monomers, and galactose modification is another example of biomimetic targeting using simple sugar ligands. Galactosylation was performed on dextran-retinal nanogels for cancer vaccine delivery 126. The formulation exhibited improved cell targeting, which translated to significantly improved DC maturation. With its inherent adjuvancy, this immunostimulatory nanogel platform represented a potent delivery system for anticancer vaccination. Additionally, more complex carbohydrates have been studied for their natural binding interactions with immune cells. Among these, glycans have been employed as biomimetic targeting moieties. Lewis-type (Le) glycan structures can be grafted to delivery vehicles for specific binding to DC-specific intercellular adhesion molecule-3-grabbing nonintegrin (DC-SIGN) expressed on DCs 127. In one example, liposomes were modified with targeting glycans Le^B^ or Le^X^, which result in increased binding and internalization by bone marrow-derived DCs expressing DC-SIGN 128. This glycoliposome-based vaccine could boost CD4^+^ and CD8^+^ T cell responses when the melanoma antigen MART1 was co-delivered.

### 4.3 Natural Carriers

Leveraging natural constructs for biomolecule transportation is another strategy for delivering immunostimulatory agents. By deriving nanovehicles from biological systems and loading them with immunostimulants, these delivery platforms can induce potent immune responses by targeting and interacting with specific immune cell subtypes. Additionally, because many of these carriers are either naturally occurring or easily self-assembled, their production can be readily streamlined, which enhances their translational potential.

#### 4.3.1 Virus Nanoparticles

Among the naturally occurring nanocarriers, virus-like particles (VLPs) have attracted significant attention, as they can be readily used to induce immune responses. VLPs are protein structures isolated from viruses that can inherit viral targeting capabilities and lack the presence of potentially dangerous genetic material 129. Viruses can inherently activate immune responses through repetitive surface structures and pathogen-associated molecular patterns, which often carry over to VLPs 130. Identified as exogenous, VLPs can trigger potent immunity on their own, which can greatly reduce the need for incorporating other immunostimulants. Thus, owing to their intrinsic targeting and immunogenicity, VLPs can promote better antigen delivery, boost immune responses, and enhance antigen presentation to the adaptive immune system 131.

A notable example of a VLP platform for immunomodulation is one based on the cowpea mosaic virus (CPMV), which has been shown to interact with APCs 132. In one such work, VLPs made from CPMV (CPMV-VLPs) suppressed established metastatic B16F10 melanoma and generated potent systemic antitumor immunity against the poorly immunogenic cancer cells 133. After intratracheal administration, CPMV-VLPs activated neutrophils in the tumor microenvironment and coordinated downstream antitumor immune responses. In combination with an antigenic peptide derived from the human epidermal growth factor receptor 2 (HER2) protein, CPMV-VLPs have also served as a cancer vaccine for the treatment of HER2^+^ tumors 134. Upon *in vivo* administration, the CPMV-VLP platform shows significant lymph node accumulation and potently activates APCs 135.

Rod-shaped plant viruses such as the tobacco mosaic virus (TMV) have also been investigated. For example, vaccination using antigen-carrying TMV-VLPs has demonstrated efficacy against various tumor models 136. TMV-VLPs have been found to participate in specific interactions with DCs and lymphocytes and can effectively stimulate APC activation. VLP systems based on the bacteriophage Qβ have demonstrated the ability to promote DC maturation and CTL stimulation 137. CpG ODN was loaded into Qβ-VLPs for synergistic immune activation, and the resulting formulation was shown to potently prime CTL responses and maintain memory CTL levels. Additionally, a lentivector has been engineered for specific targeting to DCs 138. The platform employed a viral glycoprotein from the Sindbis virus, enabling it to avidly bind with the DC surface protein DC-SIGN and induce cell maturation. Using OVA as a model antigen, the engineered lentivector promoted production of a high frequency of OVA-specific CD8^+^ T cells after subcutaneous administration in a murine model. VLPs derived from other virus sources, such as human papillomavirus 139, 140, enterovirus 71 141, 142, and hepatitis B 143, 144, have also been evaluated for cancer immunotherapy applications.

#### 4.3.2 Protein Nanoparticles

**Figure 5:**
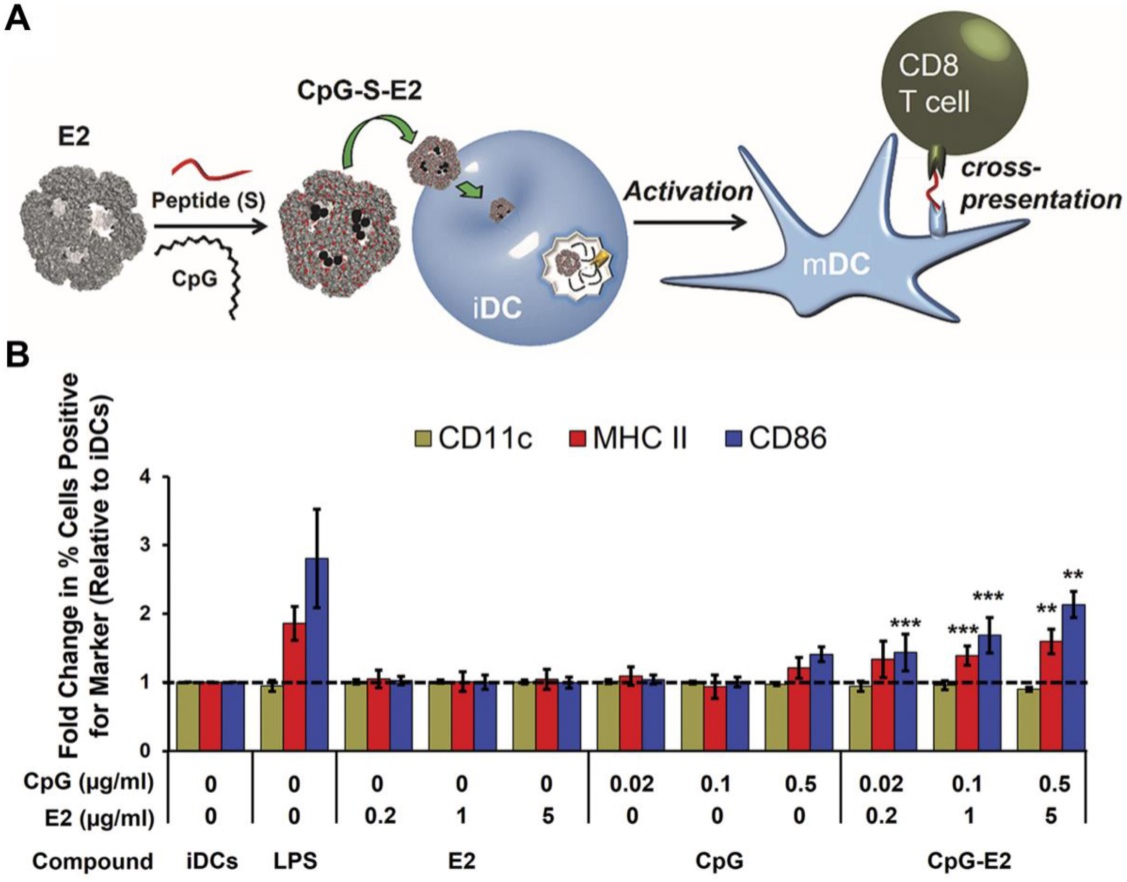
Adjuvant and antigen delivery using protein-based nanoparticles. (A) CpG ODN and a peptide antigen can be encapsulated into E2 protein nanoparticles for use as an anticancer vaccine formulation. Upon delivery into immature DCs (iDCs), they can promote transition into a mature phenotype (mDC) and enhance antigen cross-presentation to T cells. (B) The CpG-loaded E2 protein nanoparticles enhance dendritic cell maturation. Adapted with permission from 148. Copyright 2013 American Chemical Society.

Protein-based nanoparticles can be obtained by the self-assembly of protein structures from sources other than viruses 145. These particles exhibit highly-ordered surface patterns and geometries, which make them competitive delivery platforms for cancer immunotherapy applications 146. Nanoparticles assembled from the E2 component of pyruvate dehydrogenase have become an emerging class of nanocarriers for biomimetic delivery 147. Because of their small size, E2 nanoparticles are well-suited for lymphatic transport and DC uptake. Systematic work on the utilization of E2 nanoparticles as biomimetic carriers for cancer immunotherapy have been published. In one work, a virus-mimicking DC-targeted vaccine platform was engineered to deliver the DC-activating CpG ODN (**Figure 5**) 148. By co-delivering a peptide epitope from OVA along with the adjuvant using the E2 nanoparticle, DC maturation and antigen cross-presentation were achieved after particle uptake by DCs. Impressively, CpG ODN in the E2 formulation could activate DCs at a 25-fold lower concentration than free CpG ODN, which highlights the high delivery efficiency of this approach. Ultimately, the formulation was able to increase and prolong antigen-specific CD8^+^ T cell activation. In subsequent works, a variety of TAAs have been successfully delivered together with CpG ODN using E2 nanoparticles for cancer vaccination 149, 150.

Heat-shock proteins (HSPs) have also been explored for use in nanoformulations for cancer immunotherapy 151. Protein nanoparticles derived from HSPs can exhibit strong receptor-specific interactions with APCs, which facilitates downstream antigen presentation and immune stimulation 152. Several *in vivo* studies have been conducted on the use of HSP nanoparticles for immunization applications. For example, antigenic peptides bound to HSP96 have been used as cancer vaccines for patients with recurrent glioblastoma multiforme and colorectal liver metastases 153, 154. Similarly, immunization with natural HSP110 complexed with the melanoma-associated antigen gp100 protected mice against subsequent challenge with gp100-expressing B16 melanoma by bolstering both CD4^+^ and CD8^+^ T cell populations 155.

Other protein nanoparticles that have been used as natural carriers for antigen delivery include ferritin and protein vault nanoparticles. Other than their applications in drug delivery and imaging, ferritin nanoparticles were recently studied for cancer immunotherapy 156. Antigenic peptides derived from OVA were introduced to ferritin nanoparticles via attachment onto the exterior surface or encapsulation inside the interior cavity 157. Immunization with the antigen-loaded ferritin nanoparticles could efficiently induce antigen-specific CD4^+^ and CD8^+^ T cell proliferation in mice. Similarly, the inner cavity of vault nanoparticles can be used to encapsulate payloads, including immunostimulatory agents 158. For example, they were used to efficiently deliver CCL21, a lymphoid chemokine predominantly expressed in lymph nodes, in order to promote antitumor activity and inhibit lung cancer growth *in vivo* 159. Intratumoral administration of the CCL21-complexed formulation enhanced CCL21-associated leukocytic infiltrates and reduced the frequency of immunosuppressive cells.

#### 4.3.3 Lipoproteins

**Figure 6:**
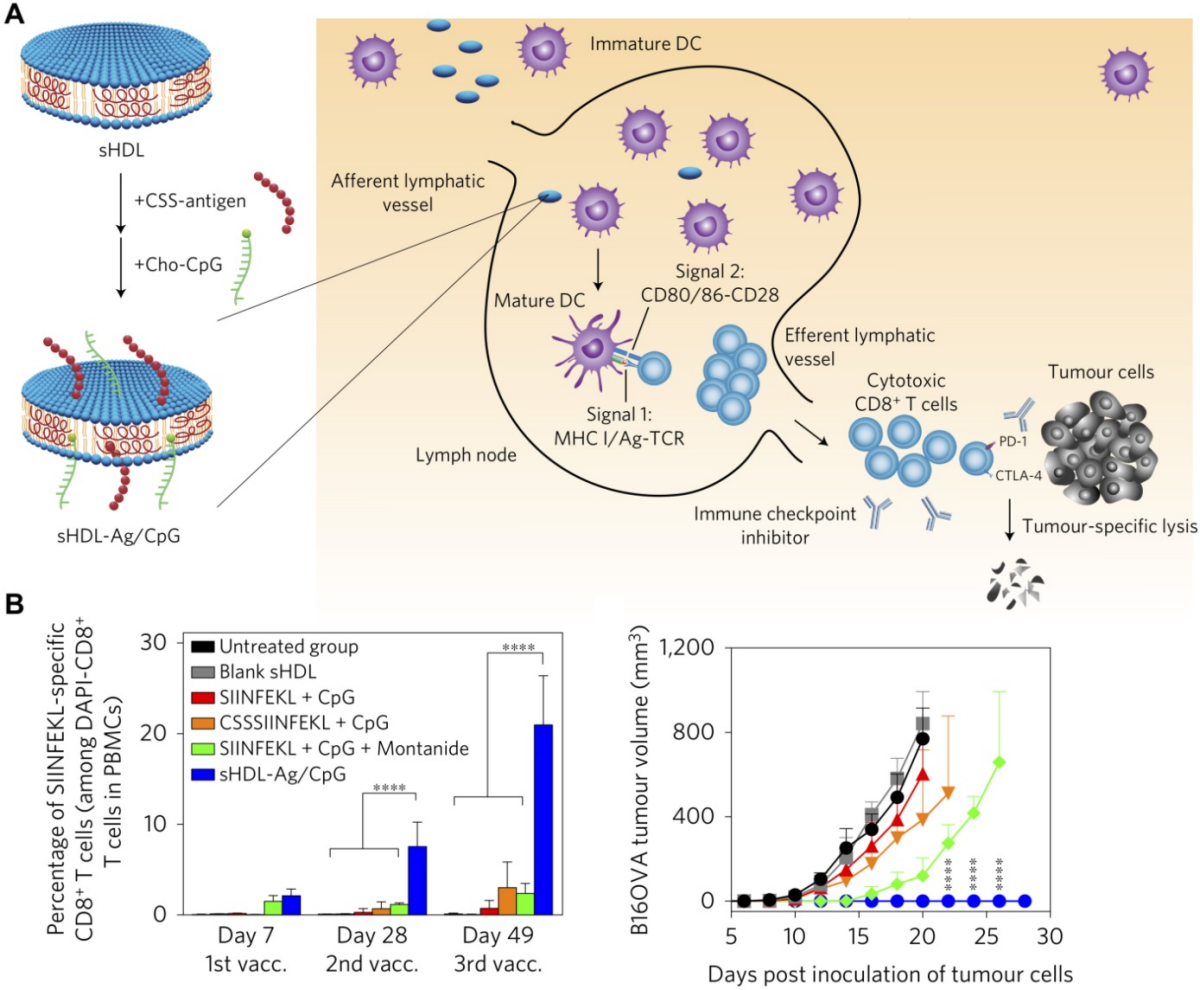
Adjuvant and antigen delivery using lipoprotein nanoparticles. (A) Synthetic high-density lipoprotein (sHDL) nanodiscs can be inserted with antigens (Ag) and adjuvants (CpG) using a cysteine-serine-serine (CSS) linker and cholesterol (Cho), respectively. Upon administration, the nanoparticles can drain into nearby lymph nodes, where they are uptaken by DCs that can subsequently activate tumor-specific T cell populations. (B) The dual-loaded nanodisc formulation elicits strong antigen-specific T cell responses and greatly inhibits tumor growth. Adapted with permission from 163. Copyright 2017 Nature Publishing Group.

Another popular type of biomimetic material that can be used for immunotherapeutic applications is lipoproteins, which are endogenous nanocarriers involved in the metabolic transport of fat molecules, as well as biomolecules such proteins, vitamins, hormones, and miRNA 160. Due to their high biocompatibility and long lifespan, lipoprotein-based nanocarriers have become emerging delivery vehicles for exogenous payload transport 161. Furthermore, the size of lipoproteins can be tuned for efficient lymph node draining and promotion of adaptive immune responses 162. Synthetic high-density lipoprotein (sHDL)-mimicking nanodiscs for personalized neoantigen vaccination and cancer immunotherapy have recently been reported (**Figure 6**) 163. In the design, cholesterol-modified CpG ODN and identified neoantigen peptides were added to the sHDL nanodiscs to prepare homogeneous ultrasmall cancer nanovaccines. The sHDL nanodiscs improved delivery to lymphoid organs and stimulated antigen presentation by DCs. Remarkably, the nanodiscs elicited a more than 30-fold greater frequency of antigen-specific CTLs compared with a soluble CpG ODN formulation, validating the robustness of using sHDL as an immunostimulant delivery platform. When combined with other immunotherapies such as anti-PD-L1 or anti-CTLA-4 mAbs, the sHDL nanodiscs could eradicate established MC-38 and B16F10 tumors *in vivo* .

Furthermore, other TLR agonists such as MPLA have been successfully incorporated into nanolipoproteins via self-assembly 164. Compared to administration of the agonist alone, its immunostimulatory profile could be significantly enhanced in the nanoformulation, resulting in elevated cytokine levels and upregulation of immunoregulatory genes. In another work, MPLA and CpG ODN were readily loaded into Ni^2+^-chelating nanodiscs via insertion into loosely packed lipid bilayers 165. His-tagged antigens were then loaded into the nanodiscs via binding to Ni^2+^. It is noteworthy that the adjuvant dosages in the nanodisc formulations were 10-fold lower than what was needed to elicit similar antibody levels and immune responses by independent administration of the components. Overall, lipoprotein-based nanocarriers represent an effective platform for antigen and adjuvant co-delivery. Additionally, it has been shown that co-delivery of chemotherapeutics along with immunostimulatory payloads via these platforms can help to further amplify antitumor efficacy 166, 167.

#### 4.3.4 Oligonucleotides and Polypeptides

Oligonucleotides can be designed to self-assemble into nanoparticles with well-defined structures and uniform sizes, and these particles have been leveraged for the delivery of immunostimulatory agents 168. In particular, CpG ODNs have been attached to structural oligonucleotides and assembled into multivalent DNA nanostructures 169. These particles were readily taken up by APCs and engaged TLR9 to activate proinflammatory immune processes. In another approach, flower-like nanostructures were self-assembled from long nucleotides integrated with tandem CpG ODNs through rolling circle replication 170. These DNA nanoparticles were able to efficiently deliver the CpG payload while preventing it from nuclease degradation. CpG-containing oligonucleotide nanostructures can also be used for the co-delivery of additional payloads. In one of such example, a programmable DNA nanocomplex was constructed through the self-assembly of a model antigen streptavidin and CpG ODN with precise control over valency and spatial arrangement 171. The resulting antigen-adjuvant nanocomplex could be used to induce long-lasting antigen-specific immunity. In another work, anti-PD-1 mAbs were loaded into a CpG ODN nanostructure to achieve synergistic action while reducing potential side effects 172. Similar to oligonucleotide nanoparticles, those based on polypeptides have also been tested for the delivery of immunostimulatory payloads. In one representative work, CpG ODN was conjugated onto polyglutamic acid, and microparticles were obtained through infiltration of the conjugates into porous silica templates, followed by crosslinking of the polypeptide chains and subsequent template removal 173. The formulation was used to successfully deliver CpG ODN to primary human DCs.

#### 4.3.5 Cell Membrane Vesicles

The last major class of naturally occurring delivery vehicles is cell membrane vesicles. Payload delivery using cell-derived membrane vesicles enables concurrent use of multiple membrane biomolecules and biomarkers for functions such as immune cell targeting, cytosolic localization, and elicitation of cytokine production, among others 115. Exosomes are fragmented vesicles secreted from cells and have essential roles in cellular signaling and metabolic transport 174. Depending on their origin, they can exhibit natural affinity towards specific tissues within the body. In the presence of proper immune stimulation, tumor cell-derived exosomes containing TAAs can induce strong adaptive immunity when delivered to APCs 175. For instance, CpG ODN was incorporated onto exosomes derived from modified B16BL6 cells 176. The CpG ODN-carrying exosomes were effective at inducing maturation of DCs for enhanced TAA presentation and generation of B16BL6-specific CTLs. Immunization with the modified exosome vaccine resulted in stronger *in vivo* immunotherapeutic efficacy on B16BL6-challenged mice compared with the co-administration of exosomes and CpG ODN. Tumor membrane has also been utilized for antigen inclusion and adjuvant delivery in a different type of approach 177. In the example, OVA-expressing B16F10 melanoma cells were lysed and vesiculated by sonication. Lipid-conjugated PEG and cholesterol-linked CpG ODN were then loaded onto the nanoparticles via lipid insertion. The resulting tumor membrane vesicle-based formulation exhibited effective lymph node draining and induced the generation of OVA-specific CTLs. When combined with anti-PD-L1 immunotherapy, the treatment mediated complete tumor regression in more than half of the animals that were treated and protected all survivors against a subsequent tumor cell re-challenge. Adjuvant loading can also be achieved by incorporation into tumor membrane particles both before and after vesiculation. In an example, whole B16F10 melanoma cells were broken down into membrane-enclosed vesicular compartments by extrusion or sonication in the presence of CpG ODN, followed by incubation with MPLA 178. The breadth and diversity of the TAA repertoire was maintained on these membrane particles. The formulation promoted the uptake of the loaded adjuvant payloads and potentiated DC activation. When administered *in vivo*, the adjuvant-loaded particles stimulated antigen-specific cellular and humoral immune responses against B16F10.

Unlike membrane vesicles from tumor origins, those derived from innate immune cells can be directly leveraged for downstream immune stimulation. For instance, membrane vesicles derived from DCs primed with tumor vesicles have been shown to activate T cells and promote robust antitumor immunity 179. In another example, immature DCs separated from C57BL/6 mice were pretreated and stimulated by the TLR4 agonist MPLA, which led to the elevated expression of costimulatory markers 180. DC membrane vesicles were then obtained after multiple freeze-thaw cycles. A model antigenic peptide from OVA was loaded into the membrane vesicles, and the resulting formulation was shown to activate immature DCs *in situ* and augment the expansion of antigen-specific CD8^+^ T cells.

Bacterial outer membrane vesicles (OMVs) have also been explored for cancer immunotherapy applications. OMVs are lipid vesicles released from the outer membrane of Gram-negative bacteria and serve a variety of roles during infection 181. They contain a number of natural adjuvants such as LPS, flagellin, and peptidoglycan that can be used to trigger strong immune reactions 182. This intrinsic immunostimulatory property has been tested in different disease applications 183. The potential of *Escherichia coli* OMVs as an effective anticancer agent has been explored, where they were tested against four different tumor models (CT26, MC38, B16BL6, and 4T1) 184. Intravenous administration of the OMVs led to accumulation in tumor tissue and induced cytokine production that enabled the growth of established tumors to be controlled.

#### 4.3.6 Genetically Modified Membrane Vesicles

In addition to their ability to encapsulate and deliver immunotherapeutic payloads, natural membrane vesicles can be genetically modified to introduce additional functionalities. IL12 plays an important role in the activation of NK cells and CTLs 185. However, the direct administration of IL12 can cause severe adverse effects, which undermine its benefits in cancer immunotherapy applications 186. In one work, cells were genetically modified to express functional IL12 using a glycolipid anchor 187. The anchored IL12 could then be efficiently intercalated and transferred onto membrane vesicles isolated from various tumor cell lines. It was found that the incorporation of IL12 onto the tumor membrane vesicles could significantly induce T cell proliferation and the release of IFNγ. In a subsequent work, together with IL12, glycolipid-anchored HER2 and CD80 were also transferred to plasma membrane vesicles homogenized from tumor tissues 188. The IL12 and CD80 served to enhance immune stimulation against the HER2 antigen. Immunization with these vesicles induced strong HER2-specific immune responses and resulted in complete protection against HER2^+^ tumor challenge.

In another type of approach, the engineering of membrane vesicles to express immunoregulatory proteins can be used to achieve a checkpoint blockade effect for antitumor therapy. In one work, PD-1 was stably expressed on the membrane of HEK 293T cells, which were subsequently extruded to form nanovesicles 189. The resulting PD-1-presenting membrane vesicles could effectively bind to and neutralize the PD-L1 ligand on tumor cells, leading to the reactivation of exhausted antigen-specific CD8^+^ T cells. Furthermore, using a similar editing process, PD-1 receptors were expressed on megakaryocytes before differentiation into platelets 190. Taking advantage of the outstanding tumor targeting ability of platelets, the platelet-derived PD-1-containing membrane vesicles could be retained at the tumor site post-resection to enhance the activity of CD8^+^ T cells against residual disease.

Other protein ligands can be integrated into membrane vesicles using similar genetic modification approaches. A virus-mimetic nanovesicle was produced by expressing viral proteins in mammalian cells, which were then sonicated in the presence of surfactants 191. This approach enabled the display of functional polypeptides with correct conformations and could aid in future vaccine design. In a different type of example, a hepatitis B virus receptor was engineered into nanovesicles in order to generate nanoscale decoys that could block infection by the virus *in vivo* 192. Besides viral proteins, tumor-targeting moieties, such as human epidermal growth factor or anti-HER2 affibodies, have been successfully integrated onto nanovesicles 193. The engineered liposome-like nanovesicles could be used to enhance the delivery of phototheranostic or chemotherapeutic agents to tumor cells.

In terms of bacterial vesicles, OMVs can also be easily modified to introduce additional functional components. As an example, *E. coli* OMVs were genetically decorated with two epitopes present in B16F10 melanoma cells expressing epidermal growth factor receptor variant III, and the resulting formulation was tested for its protective activity against tumor growth 194. High levels of antigen-specific antibody titers were elicited, and significant amounts of tumor-infiltrating lymphocytes were found at the tumor site. This ultimately led to effective protection of the immunized mice upon tumor challenge.

### 4.4 Engineered Cell Membrane Hybrids

For payload delivery, naturally occurring membrane can be integrated with other synthetic materials in a manner that takes advantage of the distinct strengths of each component. Specifically, for the delivery of immunostimulants, the presence of cell membrane-derived functionality can facilitate targeting to immune cells and accumulation in immune-rich organs, while other components can be included to augment immune stimulation performance. The membrane component can be further engineered to confer exogenous functional moieties, including cytokines, receptor-binding ligands, targeting antibodies, and immunogenic antigens, among others 195. Compared with traditional nanoformulations, a major advantage of these hybrid platforms is the ability of the natural component to camouflage artificial materials that would normally be cleared quickly by the immune system 196. These approaches also enable sophisticated delivery strategies where different payload combinations can be employed in unique ways 197. Additionally, in these hybrid systems, the intrinsic properties of various synthetic nanomaterials can be readily leveraged to achieve multimodal functionality or to create combinatorial treatments 115.

#### 4.4.1 White Blood Cell Membrane Hybrids

Mimicking the function of immune cells can be an effective means for achieving targeted delivery of immunostimulatory agents for cancer therapy. The transfer of bioactive cellular components to synthetic particles is one of the strategies that can bestow the biological functions of immune cells to synthetic hybrids 198. A bottom-up approach has been proposed based on the extraction of plasma membrane proteins from macrophages and subsequent incorporation of these proteins with synthetic choline-based phospholipids 199. The assembled hybrid vesicles retained the targeting capability of macrophages and were used for preferential targeting to inflamed vasculature. Similarly, porous silicon particles have been cloaked using membrane derived from leukocytes 200. The resulting hybrid particles possessed immunological functionalities similar to the source cells, including protection from opsonization, reduced phagocytic uptake, and binding to tumor endothelium. It has been shown that the source of membrane is critical for improving systemic tolerance and minimizing inflammatory responses 201. Membrane hybrid particles derived from syngeneic membrane exhibited less uptake by the murine immune system compared with those fabricated from xenogeneic membrane, possibly due to the presence of critical biomarkers and self-recognition receptors preserved after cloaking.

A recent work described the coating of leukocyte membrane onto magnetic nanoclusters for the construction of artificial APCs 202. Specifically, a macrophage cell line was pre-modified with azide before membrane extraction and uniformly coated onto the nanocluster cores. The nanohybrids were then functionalized with an MHC complex and anti-CD28 for antigen presentation to CD8^+^ T cells. The resulting artificial APCs could not only stimulate the expansion of antigen-specific CTLs, but also helped to effectively guide reinfused CTLs to tumor tissues through magnetic control. Immunotherapeutic nanoformulations cloaked by membrane from another leukocyte cell type, NK cells, have also been reported 203. NK cells were selected because of their immunoregulatory roles. By coating polymeric nanoparticles with NK cell membrane, the resulting particles were able to induce M1 macrophage polarization and elicit tumor-specific immune responses. A photosensitizer was loaded into the polymeric cores for photodynamic therapy, which helped to improve immunotherapeutic efficacy of the system by inducing expression of damage-associated molecular patterns on dying tumor cells.

#### 4.4.2 Red Blood Cell Membrane Hybrids

Owing to their high blood abundancy, facile processing, and remarkable biocompatibility, red blood cells (RBCs) have used extensively as a source of membrane coating material to construct versatile platforms for nanodelivery applications 204, 205. The resulting membrane-coated nanoparticles can protect encapsulated payloads from immune clearance and facilitate enhanced delivery. As recently discovered, RBCs can help to mediate certain immune processes 206, 207, which may eventually be leveraged for immunotherapeutic applications. Their ability to interact with certain pathological immune cell subsets has also aided in the design of targeted membrane-coated nanoformulations 208. In the work, a subpopulation of B cells was positively labelled by RBC membrane-coated nanoparticles based on cognate receptor binding. Additionally, an active particulate vaccine system based on RBC membrane-coated micromotors has recently been reported 209. Antigen-inserted RBC membrane was integrated with core-shell micromotors that provided propulsion properties for enhanced oral vaccination. The RBC membrane-coated vaccine formulation demonstrated improved retention in the mucosal layer of the small intestine, which led to more robust antibody production.

Specifically in terms of cancer applications, an RBC membrane-based nanovaccine platform for the stimulation of antitumor immunity was recently reported 210. The platform was constructed by enveloping RBC membrane around a polymeric PLGA core, which was used to load MPLA adjuvant and an antigenic peptide. Additionally, mannose was inserted into the RBC membrane for active APC targeting. Enhanced retention in the draining lymph nodes after intradermal injection was observed, along with elevated IFNγ secretion and CD8^+^ T cell responses. This nanovaccine effectively inhibited tumor growth and suppressed tumor metastasis in a murine B16F10 melanoma model.

#### 4.4.3 Cancer Cell Membrane Hybrids

Cancer cell membrane represents a rich source of functional ligands as well as TAAs 115, 116, and these properties have been leveraged in the design of hybrid nanostructures for cancer imaging 211, photothermal therapy 212, photodynamic therapy 213, virotherapy 214, and immunotherapy 215. In one such work on cancer immunotherapy, the immunogenic properties of HSP70 was leveraged to enhance immune responses against cancer cell membrane antigens 216. The protein was incorporated into a membrane structure along with TAAs from B16-OVA cell membrane, which was subsequently coated around a phosphate calcium core encapsulating CpG ODN. The platform effectively delivered the antigen and adjuvant payloads to APCs and NK cells, which led to the expansion of IFNγ-expressing CD8^+^ T cells and NKG2D^+^ NK cells. In another approach, the membrane from MDA-MB-231 breast cancer cells was coated around thermally oxidized porous silica, which was used as a novel immunostimulatory agent 217. The resulting hybrid nanoparticles greatly enhanced IFNγ secretion by peripheral blood monocytes and oriented the polarization of T cells towards a T_h_1 phenotype.

**Figure 7:**
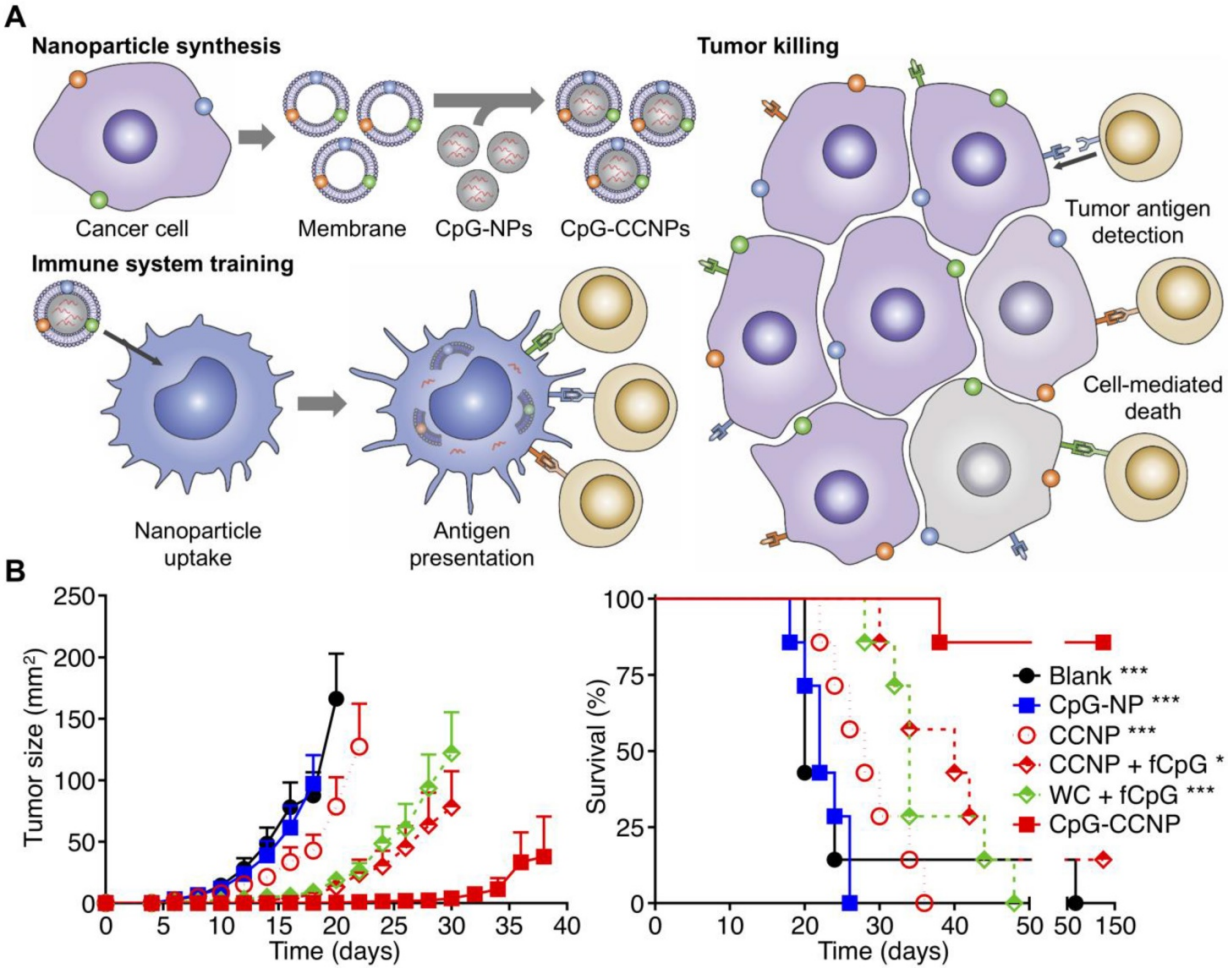
Anticancer vaccination using cancer cell membrane-coated nanoparticles (CCNPs). (A) The membrane derived from cancer cells, along with its associated tumor antigens, is coated onto CpG ODN-loaded nanoparticle cores to yield a nanoparticulate anticancer vaccine (CpG-CCNPs). Upon delivery to APCs, the vaccine formulation enables activation of T cells with multiple antitumor specificities. (B) The co-delivery of both tumor antigens and CpG together in CpG-CCNPs greatly protects against tumor growth and enhances survival. Adapted with permission from 220. Copyright 2017 Wiley-VCH.

**Figure 8:**
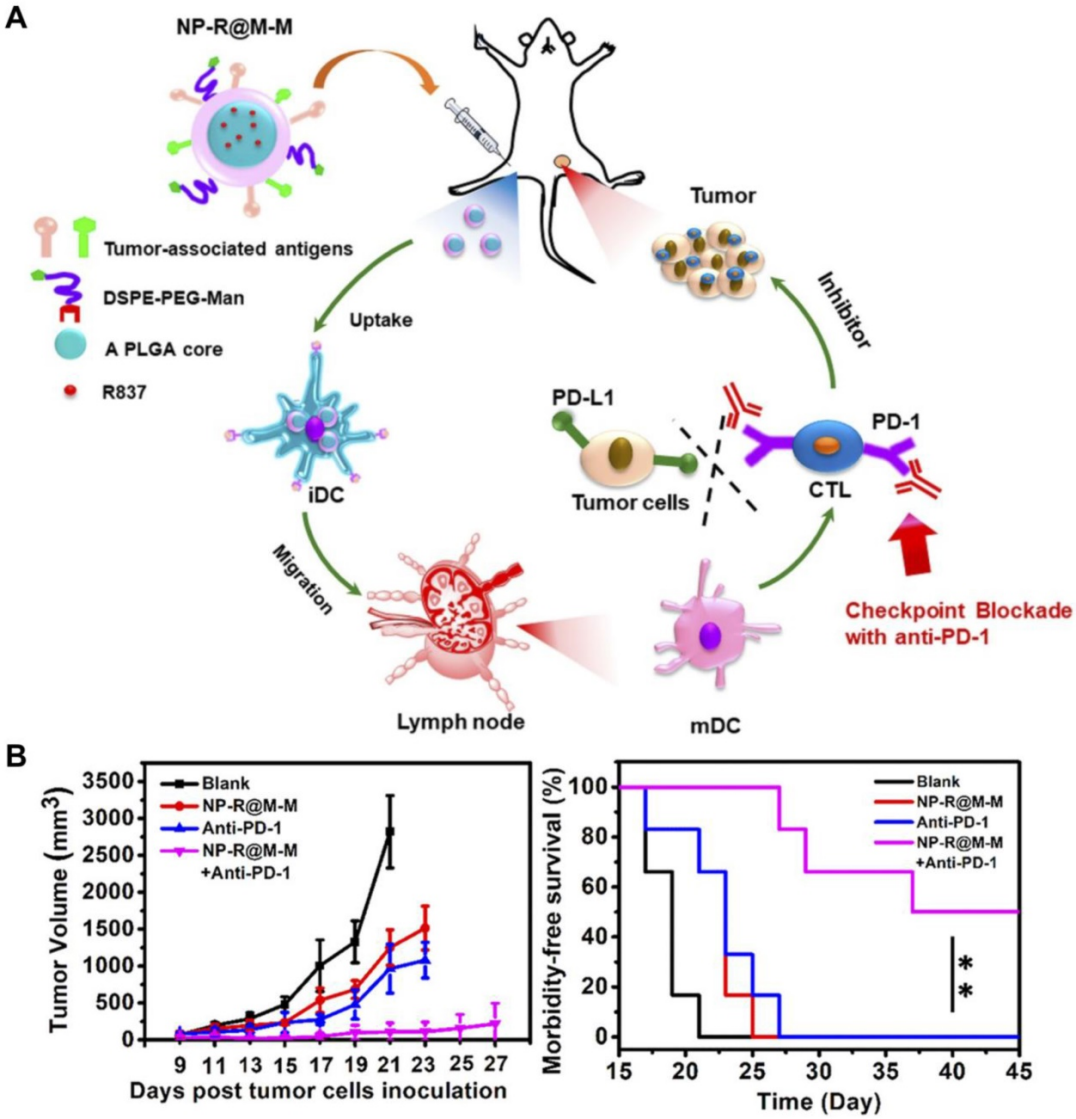
Anticancer vaccination using targeted CCNPs. (A) Tumor cell membrane-coated, R837-loaded, and mannose-modified PLGA nanoparticles (NP-R@M-M) can promote transition of DCs from an immature (iDC) to mature (mDC) phenotype. (B) When combined with checkpoint blockade therapy, tumor growth can be effectively inhibited, and survival is enhanced. Adapted with permission from 221. Copyright 2018 American Chemical Society.

Without the assistance of immunostimulatory agents, the immunogenicity of TAAs is generally insufficient to elicit potent antitumor responses 218. In addition to the above examples, there are many other strategies by which adjuvants can be included in cancer cell membrane-based nanoformulations. In an example, cell membrane from B16F10 melanoma coated onto PLGA nanoparticles was incorporated with the adjuvant MPLA 219. Besides its ability to homotypically target the source cancer cells, this cell membrane hybrid platform could efficiently induce the maturation of professional APCs and improved downstream T cell stimulation. In a follow-up study, CpG ODN loaded into PLGA cores was used to generate another anticancer vaccine formulation (**Figure 7**) 220. The nanoparticulate delivery of the adjuvant significantly enhanced its biological activity compared with CpG ODN in free form. Upon uptake by DCs, the nanovaccine formulation promoted the generation of multiple CTL populations with tumor specificity. When combined with other immunotherapies such as checkpoint blockades, the nanoformulation demonstrated the ability to significantly enhance control of tumor growth in a therapeutic setting. Over time, increasingly sophisticated nanovaccine formulations have been developed using the membrane coating concept. In a recent design, PLGA nanoparticles were loaded with the TLR7 agonist R837 and then coated with membrane from B16-OVA cancer cells (**Figure 8**) 221. To provide APC targeting functionality, the membrane shell was further modified with a mannose moiety using a lipid anchoring approach. The hybrid nanoformulation not only exhibited efficacy in delaying tumor growth as a preventative vaccine, but also displayed activity against established tumors when co-administered with anti-PD-1 mAbs.

## 5. Conclusions and Perspectives

In this review, we have discussed current progress in the development of nanoscale platforms for the delivery of immunostimulatory agents. Adjuvants, cytokines, and mAbs all represent immunotherapeutic agents that can benefit from the enhanced transport afforded by nanodelivery. The formulation of these compounds into particulate nanocarriers protects their biological activity and elevates their bioavailability, both of which can contribute to stronger immune stimulation. To address the need for specific delivery to target immune cell subsets and immune-rich tissues, bioinspired platforms and modifications can provide certain advantages over current nanoparticle technologies. Biomimetic delivery approaches generally enable facile immune cell targeting, and the inherent immunogenicity or antigenicity associated with many of these platforms can be directly leveraged for more efficient vaccine design. Furthermore, by integrating immunostimulants with tumor antigens in the same particulate system, significant immunotherapeutic efficacy against established tumors can be achieved.

Although the emerging biomimetic approaches discussed in this review have shown significant potential for cancer immunotherapy, there are still several areas in which improvements can be made. For one, further enhancement of immunostimulatory potency in a safe manner is highly desirable. This can be achieved by improving targeting efficacy or developing new materials with better immunostimulatory characteristics. As tumor immunosuppression occurs by a variety of different mechanisms, it is likely that a large percentage of patients will not respond to mono-immunotherapies. Therefore, effort will need to be placed on the exploration of how to best combine different immunotherapeutic modalities to maximize antitumor responses. For example, agents that affect innate and adaptive immunity can be combined together to provide comprehensive immune activation. Otherwise, immunotherapies can also be combined with other therapeutic modalities, including surgery, radiation, chemotherapy, and targeted therapy, among many others. Finally, as biomimetic technologies mature, more work will need to be done in order to facilitate clinical translation. Challenges along these lines include the cost-effective sourcing of biological nanomaterials, large-scale production of pharmaceutical grade products, and optimization of long-term storage conditions. As many of these promising new platforms exist at the interface between natural and synthetic, this is a new frontier that will need to be explored in concert with regulatory agencies.

## Figures and Tables

**Table 1:** Biomimetic strategies for the nanodelivery of immunostimulatory agents.

Strategy	Key points	Examples
Biomimetic modifications	Direct modification of traditional nanocarriers. Facile and controllable processes.	Simple sugars^123^, ^124^, ^126^
		Glycans^128^
Natural carriers	Adaptation of natural carriers from biological systems. Straightforward collection, derivation, or self-assembly. Natural immune stimulation or targeting properties. High biocompatibility.	Virus nanoparticles^133^, ^137^, ^138^
		Protein nanoparticles^148^, ^150^, ^153^, ^157^, ^159^
		Oligonucleotides/polypeptides^169^-^173^
		Lipoproteins^163^-^165^
		Cell membrane vesicles 176-178, 180, 184
		Genetically modified vesicles^187^, ^189^, ^193^, ^194^
Cell membrane hybrids	Combination of naturally occurring and synthetic nanomaterials. Natural immune stimulation or targeting properties. Multimodal functionality.	White blood cell hybrids^200^, ^202^, ^203^
		Red blood cell hybrids^208^-^210^
		Cancer cell hybrids^216^, ^217^, ^219^-^221^
